# Bioinformatics-led discovery of ferroptosis-associated diagnostic biomarkers and molecule subtypes for tuberculosis patients

**DOI:** 10.1186/s40001-023-01371-5

**Published:** 2023-10-19

**Authors:** Dilinuer Wufuer, YuanYuan Li, Haidiya Aierken, JinPing Zheng

**Affiliations:** 1 The First Affiliated Hospital of Guangzhou Medical University/National Clinical Research Center for Respiratory Disease/National Respiratory Medical Center/State Key Laboratory of Respiratory Disease/Guangzhou Institute of Respiratory Health, NO. 151 Yanjang Road, Guangzhou, 510120 China; 2 Department of Respiratory Medicine, Eighth Affiliated Hospital of Xinjiang Medical University, Urumqi, 830049 Xinjiang China; 3 Department of Respiratory Medicine, First Affiliated Hospital of Xinjiang Medical University, Urumqi, 830054 Xinjiang China

**Keywords:** Tuberculosis, Ferroptosis, Diagnostic biomarkers, xCell, Molecular types

## Abstract

**Background:**

Ferroptosis is closely associated with the pathophysiological processes of many diseases, such as infection, and is characterized by the accumulation of excess lipid peroxides on the cell membranes. However, studies on the ferroptosis-related diagnostic markers in tuberculosis (TB) is still lacking. Our study aimed to explore the role of ferroptosis-related biomarkers and molecular subtypes in TB.

**Methods:**

GSE83456 dataset was applied to identify ferroptosis-related genes (FRGs) associated with TB, and GSE42826, GSE28623, and GSE34608 datasets for external validation of core biomarkers. Core FRGs were identified using weighted gene co-expression network analysis (WGCNA). Subsequently, two ferroptosis-related subtypes were constructed based on ferroptosis score, and differently expressed analysis, GSEA, GSEA, immune cell infiltration analysis between the two subtypes were performed.Affiliations: Please check and confirm that the authors and their respective affiliations have been correctly identified and amend if necessary.correctly

**Results:**

A total of 22 FRGs were identified, of which three genes (CHMP5, SAT1, ZFP36) were identified as diagnostic biomarkers that were enriched in pathways related to immune-inflammatory response. In addition, TB patients were divided into high- and low-ferroptosis subtypes (HF and LF) based on ferroptosis score. HF patients had activated immune- and inflammation-related pathways and higher immune cell infiltration levels than LF patients.

**Conclusion:**

Three potential diagnostic biomarkers and two ferroptosis-related subtypes were identified in TB patients, which would help to understand the pathogenesis of TB.Author names: Kindly check and confirm the process of the author names [2,4]correctly

**Supplementary Information:**

The online version contains supplementary material available at 10.1186/s40001-023-01371-5.

## Introduction

Tuberculosis (TB) is a chronic infectious disease that poses a serious threat to human health and is considered one of the world’s three major infectious diseases, along with malaria and acquired immunodeficiency syndrome [1]. According to the World Health Organization, China had the third highest number of new cases of TB in the world with about 833,000 cases in 2019 [2]. TB is mainly caused by infection with *Mycobacterium tuberculosis* (M.tb). It is currently one of the ten leading causes of death worldwide and the leading cause of death from a single infectious disease [3, 4]. M.tb can cause infection in almost any part of the body, and the spectrum of clinical disease following M.tb infection is diverse, ranging from asymptomatic to life-threatening acute infectious disease [5, 6]. Although the utilization of various methods for diagnosing TB, the process remains challenging, especially in the absence of a clear focus of infection [7–9]. Therefore, developing new biomarkers to help diagnose active TB is urgently needed.

Ferroptosis is a non-apoptotic form of cell death, characterized by the accumulation of iron-dependent lipid peroxides to the point of cell death [10, 11]. In several diseases, including sepsis, neurodegenerative disease, diabetes, and cancer, ferroptosis has been implicated in a variety of physiological and pathological processes [12, 13]. In addition, ferroptosis has also been shown to play an important role in the pathogenesis of pulmonary disease including lung cancer, pulmonary fibrosis, chronic obstructive pulmonary disease, and pneumonia [14–16]. A recent study revealed that inhibition of ferroptosis suppressed M.tb-induced bacterial load and tissue necrosis [17]. Additionally, the exploration of ferroptosis inhibitors that target the interaction between M.tb PtpA and Ran-GDP could be pursued as a potential treatment for TB [18, 19]. Thus, ferroptosis may have a vital role in the pathogenesis of TB and that the ferroptosis-related genes (FRGs) may be involved in the occurrence of TB.

In recent years, some researchers have combined transcriptome sequencing with bioinformatics analysis to identify differentially expressed genes (DEGs) and functional pathways involved in the pathological process of pulmonary disease [20, 21]. In the present research, gene expression profiles of TB patients from the Gene Expression Omnibus (GEO) database were comprehensively analysed using bioinformatics. We aimed to gain insight into the potential pathological mechanisms of TB and to identify ferroptosis-related targets and biomarkers for the early diagnosis of TB.Article structure: Kindly check whether the section headings have been identified correctly and amend if any.correctly

## Methods and materials

### Collection of datasets

A total of four TB-related gene expression microarrays (GSE83456, GSE42826, GSE28623, and GSE34608) downloaded from the GEO database of the National Center for Biotechnology Information (NCBI, https://www.ncbi.nlm.nih.gov/). The information of the GEO datasets was presented in Table 1. The selection criteria for the datasets are as follows: the experimental design of the gene expression profiling dataset must be a whole blood study of active TB patients and healthy controls (HC); the number of samples of both types of whole blood involved in each gene expression profiling dataset must be greater than 5; the samples for the gene expression profiling study must be RNA expressed at the level of the whole human genome. The GEOquery function of the R language Bioconductor package was used to download gene expression profile data. FerrDb, the first database of experimentally validated ferroptosis regulators and markers and ferroptosis-disease associations. Annotations were generated from currently available ferroptosis articles in PubMed [22]. From the FerrDb database (http://www.zhounan.org/ferrdb), a total of 259 ferroptosis-related genes (FRGs) were downloaded.

**Table 1:** The GEO datasets information

GEO ID	Platform	Healthy control (HC)	Active pulmonary tuberculosis (TB)	Latent pulmonary tuberculosis (LTB)	Source	Application
GSE83456	GPL10558	61	45	0	Blood	Analysis
GSE42826	GPL10558	52	11	0	Blood	Validation
GSE28623	GPL4133	37	46	25	Blood	Validation
GSE34608	GPL6480	18	8	0	Blood	Validation

### Identification of FRGs

Limma, which is an R software package, offers a comprehensive solution for the analysis of gene expression data. It has gained significant popularity as a preferred tool for gene discovery through the examination of differential expression in microarray [23]. The “limma” package of R was used to screen the DEGs in the different groups based on the set cutoff criteria of p.adjust < 0.05 and | log fold change (FC)|≥ 1. The “ggplot2” was used to map the volcano plot and boxplot. Metascape is an online analysis platform that offered biologists with a comprehensive annotation and analysis resource [24]. Functional enrichment analyses were performed using Metascape to investigate the biological functions and pathways of the DEGs.

### Gene set enrichment analysis (GSEA)

GSEA is a method employed to detect gene sets that exhibit differential expression and are enriched with known biological functions [25]. For GSEA, the ClusterProfiler package was applied. The “h.all.v7.4.symbols.gmt” subset was downloaded from the Molecular Signatures Database for evaluation of the associated pathways. Statistical significance was defined as p value < 0.05.

### Weighed gene co-expression network analysis (WGCNA)

WGCNA can be used for the discovery of highly relevant gene modules, which can be used for the identification of therapeutic targets or candidate markers [26]. Based on the gene expression profile data, the median absolute deviation (MAD) was calculated for each gene separately. Then, the top 50% of genes with the lowest MAD were removed. Subsequently, goodSamplesGenes method of WGCNA package was used to remove abnormal genes and samples, and WGCNA was used to construct co-expression network. Hub genes were screened based on the cut-off criteria Module membership > 0.8 and Gene significance > 0.3.

### Immune microenvironment analysis

xCell is a powerful computational technique that transforms gene expression profiles into enrichment scores for immune and stroma cell types across various samples [27]. The xCell algorithm was used to calculate the difference in the proportion of different infiltrating immune cells in the immune microenvironment between the TB and HC, and results were shown in a box plot. In addition, the correlation between the core FRGs expression and immune cell infiltration was performed using the “ggplot2” package, and the results were shown in lollipop chart.

### Gene set variation analysis (GSVA)

The GSVA technique is characterized by its non-parametric and unsupervised nature, which eliminates the need to explicitly model phenotypes in the enrichment scoring algorithm. This approach enhances the ability to identify even subtle variations in pathway activity across different individuals [28]. In our study, the ferroptosis score was calculated using the GSVA algorithm based on the gene expression profile of FRGs. The score matrix of the ferroptosis was obtained and result was visualized by box plot.

### Identification of ferroptosis-related subtypes

TB patients were divided into the low ferroptosis score (LF) and high ferroptosis (HF) score subgroups based on the median value of the ferroptosis score. Subsequently, differential expression, GSEA, GSVA and immuno-infiltration analyses were performed between the two subgroups using the corresponding R software.

### Validation of core genes by qRT-PCR

Blood samples from 8 HC and 8 TB patients were collected from the First Affiliated Hospital of Guangzhou Medical University. The study obtained written informed consent from all participants and was approved by the Ethics Committee of The First Affiliated Hospital of Guangzhou Medical University. Blood samples were subjected to RNA extraction using the TRIzol reagent (Invitrogen) following the manufacturer’s protocol. The total RNA obtained was then reverse transcribed into complementary DNA (cDNA) using the cDNA Synthesis Kit (Takara, China). qRT-PCR was carried out with Taq Universal SYBR green Supermix (Bio-Rad, USA). The 2^−ΔΔCt^ method was used to calculate gene expression relative to GAPDH expression. Primers used were presented in Additional file 2: Table S1.Additional file: As per journal requirements, every additional file must have a corresponding caption. In this regard, please be informed that the caption of Additional file [1] was taken from the additional e-file itself. Please advise if action taken appropriate and amend if necessary.correctly

## Results

### Expression landscape of FRGs in TB

**Fig. 1:**
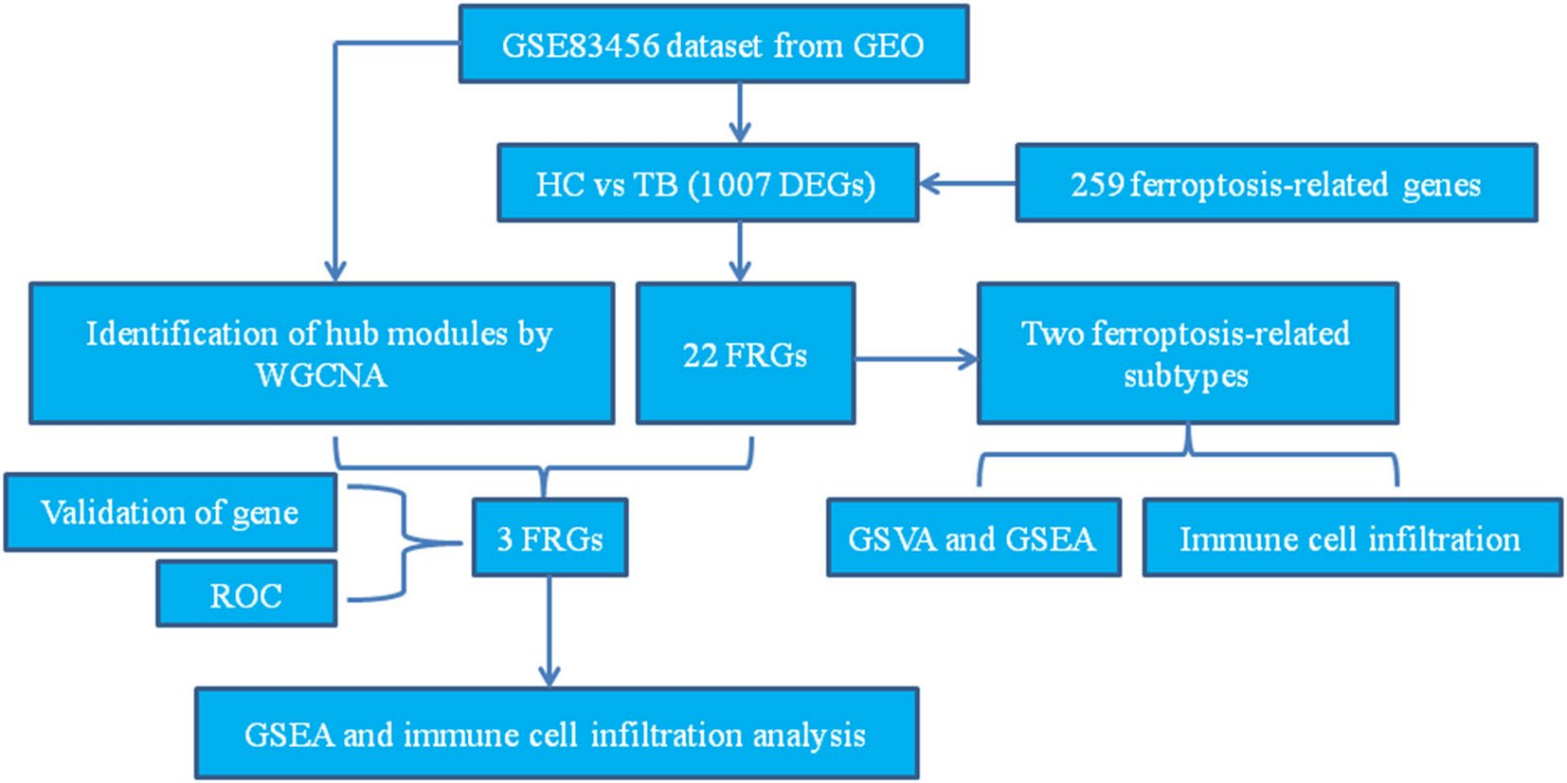
Flow chart of the data analysis process. The GSE83456 dataset was used to find FRGs associated with TB. The core FRGs were identified using WGCNA, and validated using the GSE42826, GSE28623, GSE34608 datasets and qRT-PCR analysis. Two subtypes related to ferroptosis were then created based on the ferroptosis score. Differential expression analysis, GSEA, and immune cell infiltration analysis were performed between the two subtypes

**Fig. 2:**
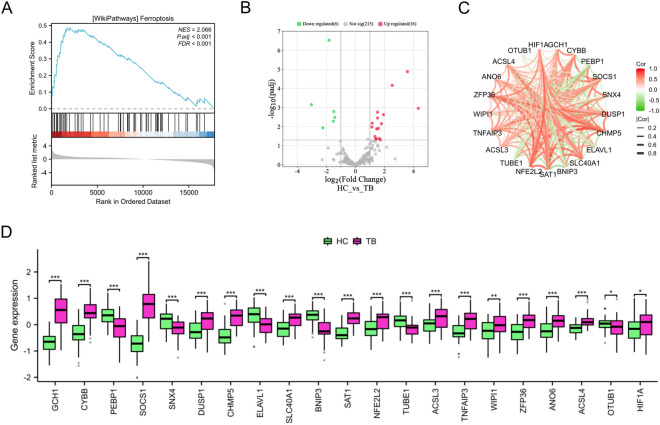
Expression profile of FRGs in TB. **A** The GSEA results suggested that ferroptosis is important in the pathogenesis of TB. **B** Volcano plot of the FRGs (the green dots represented the down-regulated FRGs and red dots represented the up-regulated FRGs). **C** The correlation plot represented the degree of correlation of the 22 FRGs. **D** Box plots depicted the differentially expressed FRGs between the HC and TB groups. *p < 0.05, **p < 0.01, ***p < 0.001

The analysis flow for this study is shown in Fig. 1. A differential study comparing samples from HC and TB revealed 1007 DEGs. Of these, 390 were found to be upregulated while 617 were downregulated (Additional file 1 Figure S1A, Additional file 2: Table S1). Functional enrichment analysis was performed using Metascape. The results, as shown in Additional file 1: Figures S1B–C, indicated that these DEGs were primarily enriched in immune-related pathways, such as response to virus, cytokine signaling in immune system, interferon signaling, adaptive immune system, alpha–beta T cell activation, etc. Additionally, according to the GSEA enrichment results, there was a significant enrichment of ferroptosis in the TB group (as shown in Fig. 2A, NES = 2.06, p.adj < 0.001). The results implied that ferroptosis plays an important role in TB development.

In this study, we examined the expression patterns of 259 FRGs (Additional file 2: Table S2) in both TB and HC samples. Our findings indicated that some of FRGs were significantly upregulated in TB samples compared to HC samples, including GCH1, CYBB, SOCS1, DUSP1, CHMP5, SLC40A1, SAT1, NFE2L2, ACSL3, TNFAIP3, WIPI1, ZFP36, ANO6, ACSL4, and HIF1A, while PEBP1, SNX4, ELAVL1, BNIP3, TUBE1, and OTUB1 were expressed at low levels in TB group (Figs. 2B, D). To investigate the interactions of differentially expressed FRGs, a correlation analysis was conducted. The findings revealed a significant correlation among these genes (Fig. 2C).

### Identification of core FRGs by WGCNA

**Fig. 3:**
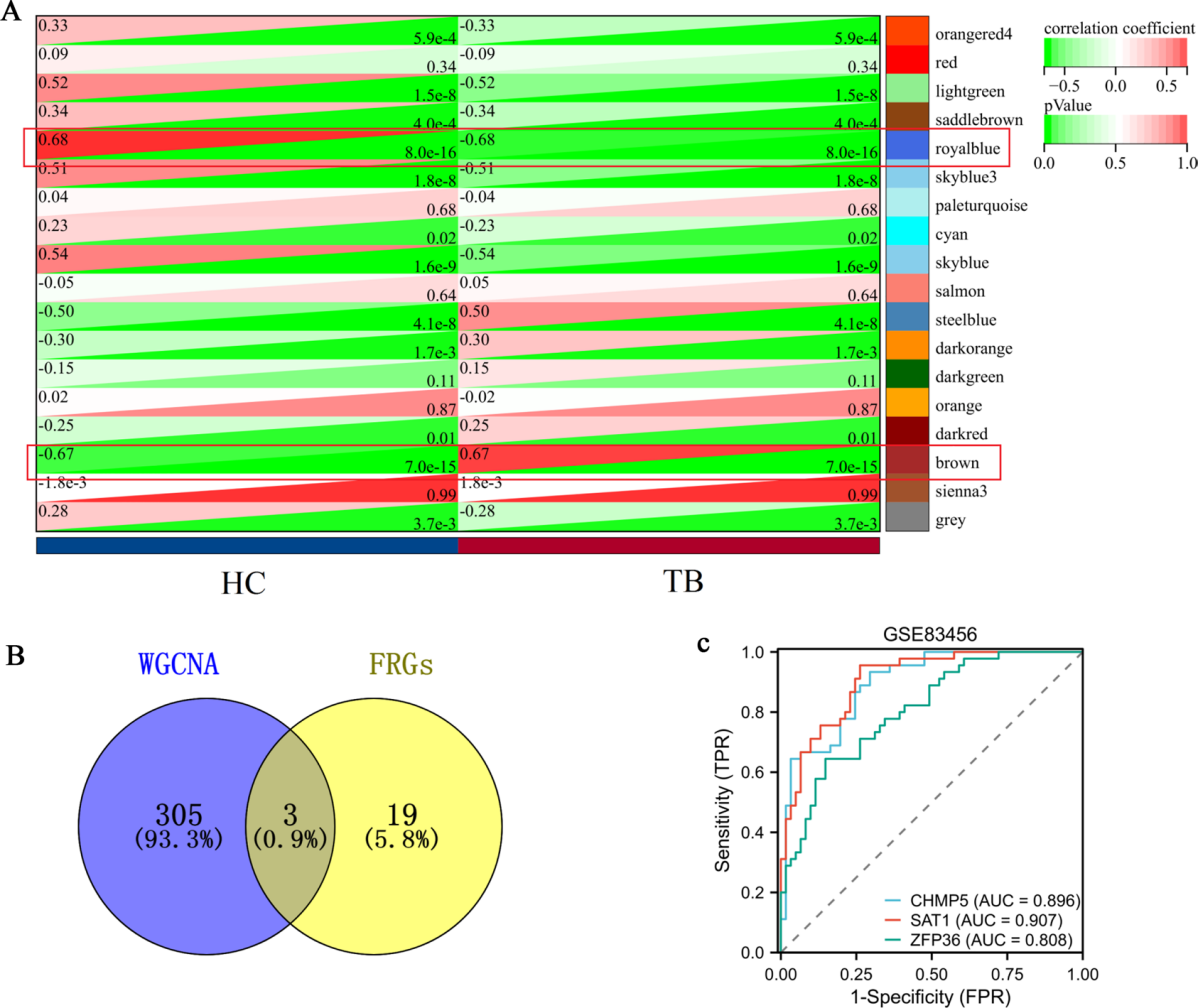
Identification of core FRGs by WGCNA. **A** Key modules identified by WGCNA. **B** The Venn diagram represented the genes that are shared between the FRGs and the WGCNA. **C** The ROC curves of CHMP5, SAT1, and ZFP36 genes in the GSE83456 dataset

We used WGCNA to identify 18 gene modules (Fig. 3A). Among these, the royalblue and brown modules showed a significant correlation with TB (r = 0.55, p = 4.2e−12 and r = 0.41, p = 1.6e−76, respectively) as depicted in Additional file 1**: **Figure S2. To further investigate, we identified 308 hub genes within these modules (Additional file 2: Table S3).

Our study identified three FRGs (CHMP5, SAT1 and ZFP36) from core modules based on the Venn result (Fig. 3B), which were found to be significantly up-regulated in the TB group (p < 0.05). Additionally, the diagnostic AUC values of CHMP5, SAT1, and ZFP36 genes were found to be 0.896, 0.907, and 0.808, respectively as depicted in Fig. 3C.

**Fig. 4:**
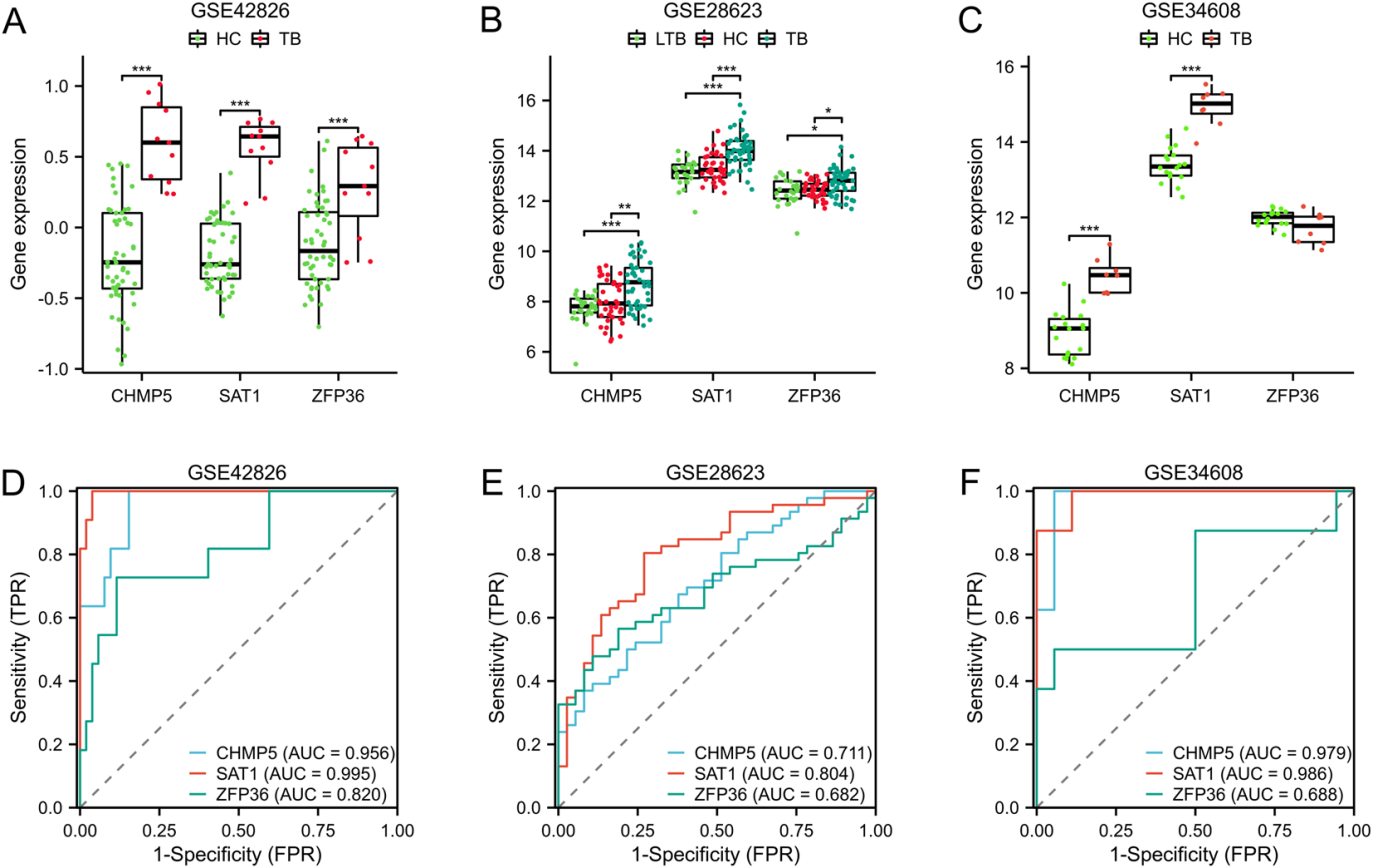
Validation of core FRGs using independent datasets. The gene expression levels **A**–**C** and ROC curves **D**–**F** of CHMP5, SAT1, and ZFP36 genes in the GSE42826, GSE28623, and GSE34608 datasets. *p < 0.05, **p < 0.01, ***p < 0.001

To validate these genes expression, multiple microarray datasets (GSE42826, GSE28623, and GSE34608 datasets) were utilized. The TB group exhibited a significantly higher expression level of CHMP5 and SAT1 genes as compared to the HC group (p < 0.01) (Fig. 4A–C). The expression of the ZFP36 gene was significantly up-regulated in the GSE42826 and GSE28623 datasets (p < 0.05). However, no significant difference was observed in ZFP36 gene expression between the normal and disease groups (Fig. 4C). ROC analysis was used to confirm the diagnostic value of core genes. In the GSE42826 cohort, the AUC values for CHMP5, SAT1, and ZFP36 genes were 0.956, 0.995 and 0.82, respectively (Fig. 4D); in the GSE28623 cohort, the AUC values for CHMP5, SAT1, and ZFP36 genes were 0.711, 0.804 and 0.682, respectively (Fig. 4E); in the GSE34608 cohort, the AUC values for CHMP5, SAT1, and ZFP36 genes were 0.979, 0.986 and 0.688, respectively (Fig. 4F).

### Investigating potential biological functions of core FRGs

**Fig. 5:**
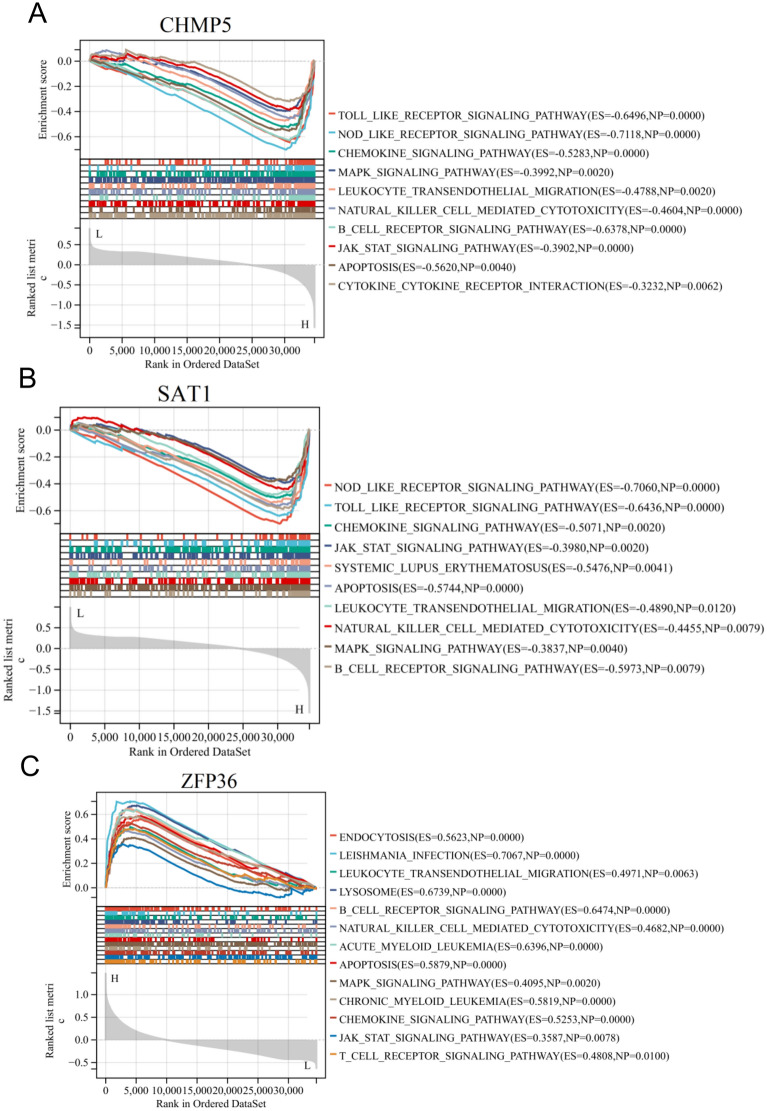
The core FRGs were analyzed using GSEA, which revealed potential signaling pathways. The immune-inflammatory pathways are significantly enriched in high expression of CHMP5 **A**, SAT1 **B**, and ZFP36 **C**

We conducted a single gene GSEA to investigate the potential biological functions of core FRGs. Our findings revealed that toll like receptor signaling pathway, chemokine signaling pathway, leukocyte transendothelial migration, natural killer cell mediated cytotoxicity, B cell receptor signaling pathway, JAK-STAT signaling pathway, and cytokine-cytokine receptor interaction were enriched in the CHMP5 high-expressed phenotype, as depicted in Fig. 5A; toll like receptor signaling pathway, chemokine signaling pathway, leukocyte transendothelial migration, natural killer cell mediated cytotoxicity, B cell receptor signaling pathway, JAK-STAT signaling pathway, and MAPK signaling pathway were enriched in the SAT1 high-expressed phenotype, as depicted in Fig. 5B; endocytosis, leishmania infection, leukocyte transendothelial migration, natural killer cell mediated cytotoxicity, B cell receptor signaling pathway, lysosome, and acute myeloid leukemia were enriched in the ZFP36 high-expressed phenotype, as depicted in Fig. 5C. The results suggested that the three FRGs may play a crucial role in regulating immune response.

### Differences in immune characteristics between the HC and TB groups

**Fig. 6:**
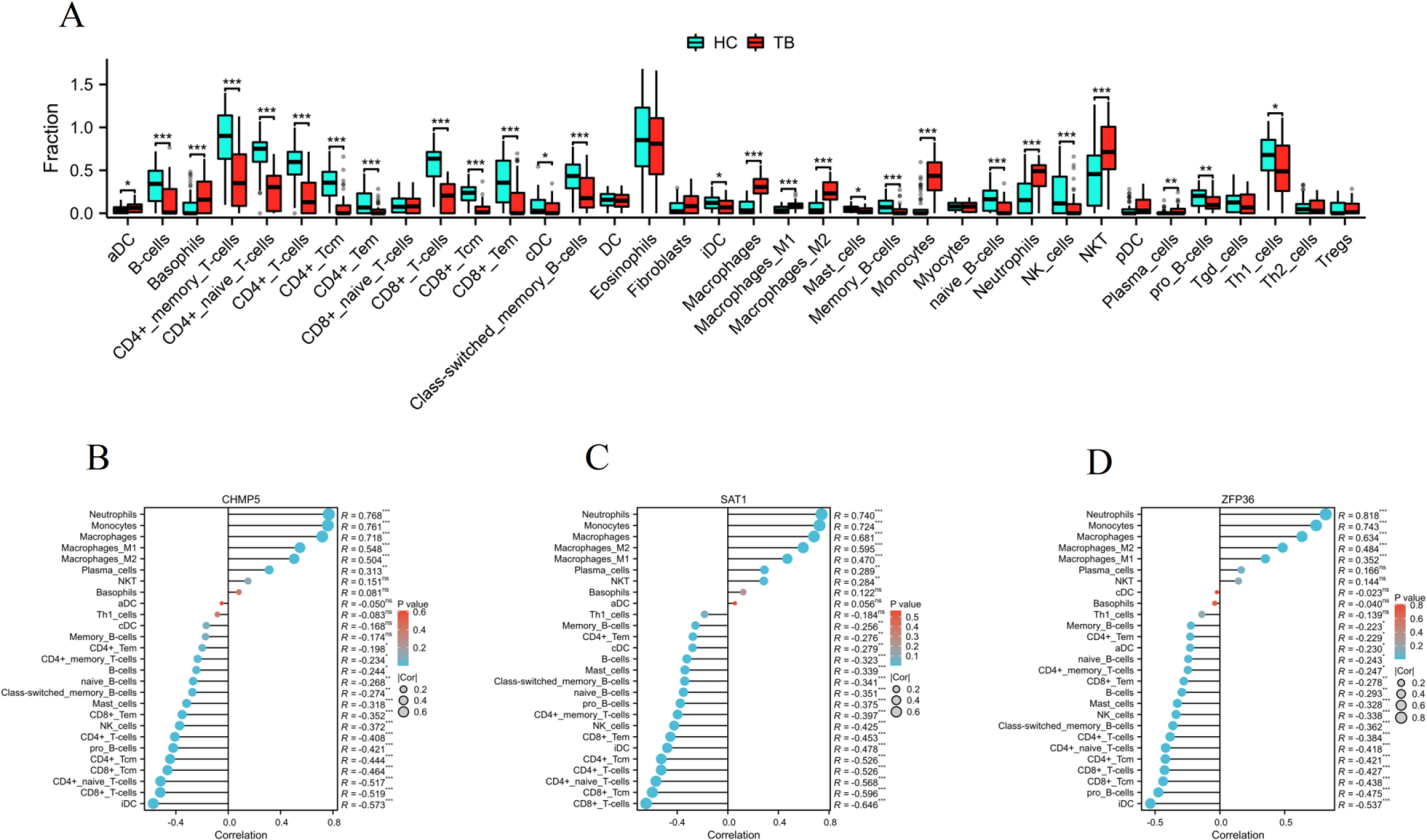
Differences in immune characteristics between the HC and TB groups. **A** Box plots depicted the landscape of immune cells infiltration between the HC and TB groups. *p < 0.05, **p < 0.01, ***p < 0.001. **B**–**D** Correlation between three core FRGs and immune cell infiltration levels. *p < 0.05, **p < 0.01, ***p < 0.001

The xCell analysis revealed significant differences in immune status between the HC and TB groups. The TB patients had significantly higher proportions of aDC, basophils, macrophages, macrophages M1, macrophages M2, monocytes, neutrophils, NKT, and plasma cells compared to the HC. However, the TB patients had significantly lower proportions of B cells, CD4 + memory T cells, CD4 + naive T cells, CD4 + T cells, CD4 + Tcm, CD4 + Tem, CD8 + T cells, CD8 + Tcm, CD8 + Tem, cDC, class-switched memory B cells, iDC, mast cells, memory B cells, naive B cells, NK cells, pro B cells, and Th1 cells compared to the HC (Fig. 6A). Our study also explored the correlation between core FRGs expression and immunological characteristics. The results depicted in Fig. 6B indicated that there was a negative correlation between CHMP5 expression and iDC, CD8 + T cells, CD4 + naive T cells, CD4 + T cells, CD8 + Tcm, CD4 + Tcm, pro B cells, CD4 + T cells, NK cells, CD8 + Tem, CD4 + Tem, while there was a positive correlation between CHMP5 expression and neutrophils, monocytes, macrophages, macrophages M1, macrophages M2, plasma cells; there was a negative correlation between SAT1 expression and iDC, CD8 + T cells, CD4 + naive T cells, CD4 + T cells, CD8 + Tcm, CD4 + Tcm, pro B cells, CD4 + T cells, NK cells, CD8 + Tem, CD4 + Tem, while there was a positive correlation between SAT1 expression and neutrophils, monocytes, macrophages, macrophages M1, macrophages M2, plasma cells, NKT (Fig. 6C); there was a negative correlation between ZFP36 expression and iDC, pro B cells, CD8 + T cells, CD4 + naive T cells, CD4 + T cells, CD8 + Tcm, CD4 + Tcm, CD4 + T cells, NK cells, mast cells, CD8 + Tem, CD4 + Tem, while there was a positive correlation between ZFP36 expression and neutrophils, monocytes, macrophages, macrophages M1, macrophages M2 (Fig. 6D).

### Identification of ferroptosis-related subtypes

**Fig. 7:**
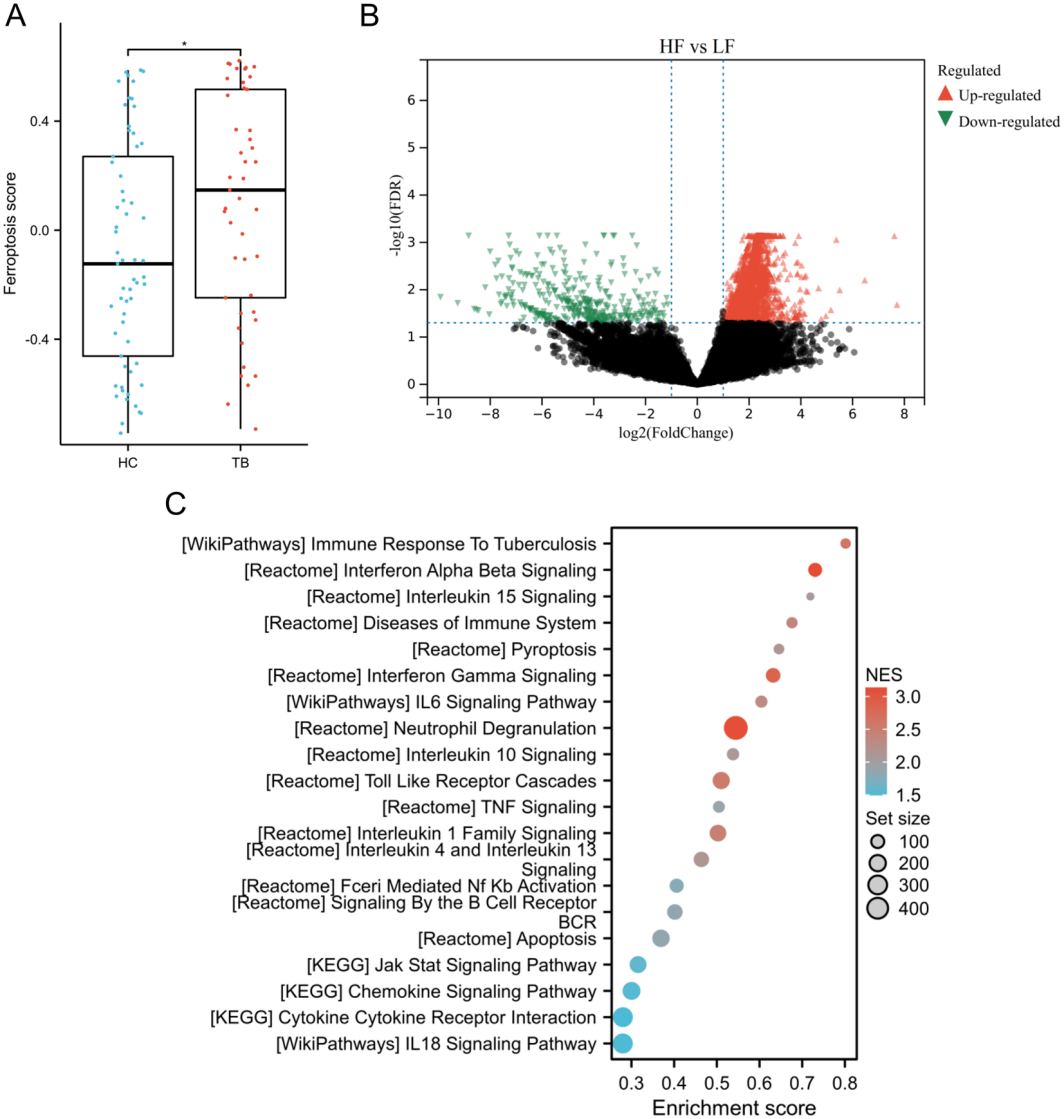
Identification of ferroptosis-related subtypes. **A** Box plots depicted the ferroptosis score between the HC and TB groups. *p < 0.05. **B** Volcano plot exhibited the DEGs between the LF and HF subgroups. **C** GSEA was performed to explore the potential pathways between the two subgroups

The GSVA algorithm was used to calculate the ferroptosis score, revealing that the TB group had a significantly higher score than the HC group (p < 0.05) (Fig. 7A). The patients with TB were separated into two subgroups: those with a low ferroptosis score (LF) and those with a high ferroptosis score (HF). The median value of the ferroptosis score was used to divide the patients into these subgroups. A differential study comparing samples from LF and HF revealed 7786 DEGs. Of these, 7406 were found to be upregulated while 380 were downregulated (Fig. 7B).

### Enrichment analysis between subtypes

GSEA results indicated that these DEGs were primarily enriched in immune-inflammatory pathways, such as immune response to tuberculosis, interferon alpha beta signaling, interleukin 15 signaling, diseases of immune system, pyroptosis, interferon gamma signaling, IL6 siganling pathway, neutrophil degranulation, interleukin 10 signaling, toll like receptor cascades, TNF signaling, interleukin 1 family signaling, apoptosis, JAK-STAT singaling pathway, chemokine signaling pathway, IL18 signaling pathway, etc. (Fig. 7C).

**Fig. 8:**
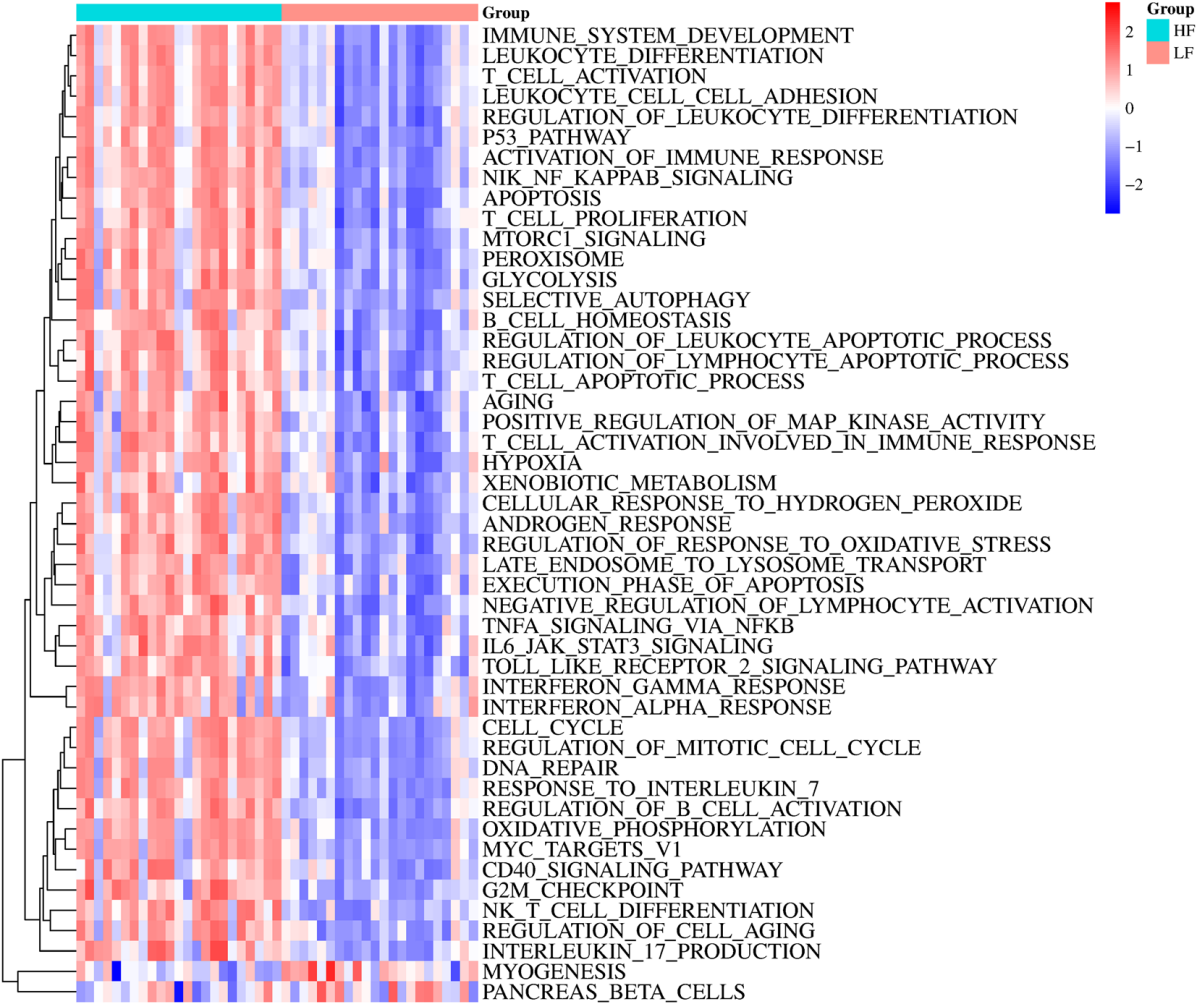
GSVA was carried out to investigate the potential pathways between the two subgroups

Additionally, we utilized GSVA to investigate the variations in participating potential pathways between the two subtypes. Our findings revealed that HF subtype exhibited a greater number of immune response relevant pathways than LF subtype, such as immune system development, leukocyte differentiation, T cell activation, leukocyte cell–cell adhesion, regulation of leukocyte differentiation, T cell proliferation, B cell homeostasis, etc. (Fig. 8).

### Immunological characteristics of both subtypes

**Fig. 9:**
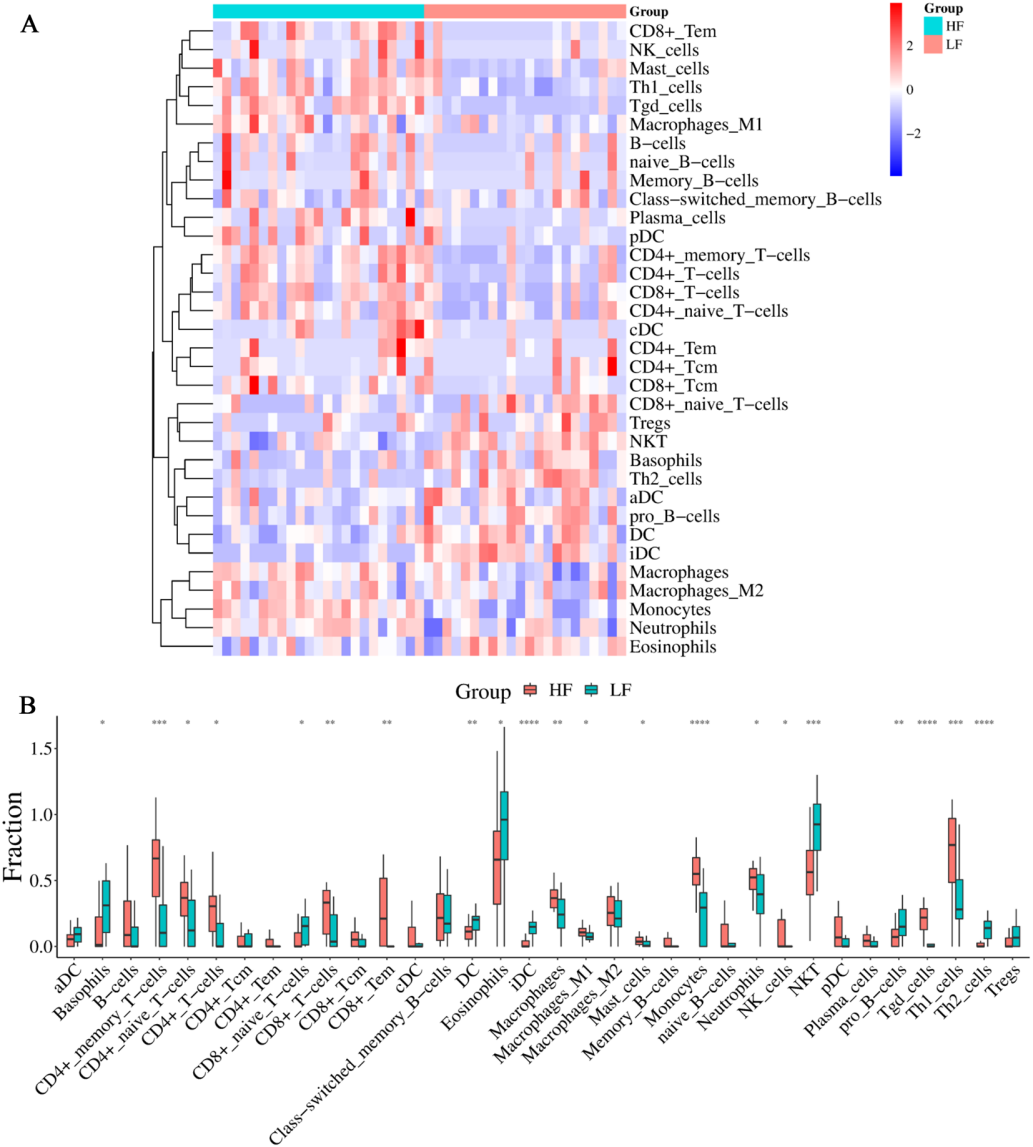
Differences in immune characteristics between the two subgroups. Heatmap **A** and box **B** plots depicted the landscape of immune cells infiltration between the LF and HF groups. *p < 0.05, **p < 0.01, ***p < 0.001

Due to the intricate nature of the immune microenvironment, it is possible for two different immune subtypes to emerge. In order to gain a better understanding of the biological behavior between these two subtypes, we utilized the xCell algorithm to analyze the proportion of immune cells present in the immune infiltrative microenvironment in TB. As shown in Fig. 9A–B, the LF subtype had significantly higher proportions of basophils, CD8 + naive T cells, DC, eosinophils, iDC, NKT, pro B cells, and Th2 cells compared to the HF subtype. However, the LF subtype had significantly lower proportions of CD4 + memory T cells, CD4 + naive T cells, CD4 + T cells, CD8 + T cells, CD8 + Tem, macrophages, macrophages M1, mast cells, monocytes, neutrophils, NK cells, Tgd cells, and Th1 cells compared to the HF subtype.

### Validation of core genes by clinical blood samples

**Fig. 10:**
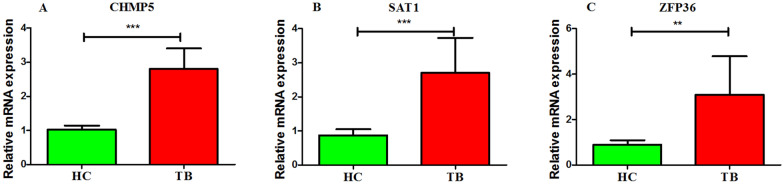
Validation of core genes by qRT-PCR. The gene expression levels of CHMP5 **A**, SAT1 **B**, and ZFP36 **C** genes in the clinical blood samples. **p < 0.01, ***p < 0.001

Clinical blood samples were collected to verify the expression levels of key genes using qRT-PCR techniques. As shown in Fig. 10, the TB group exhibited a significantly higher expression level of CHMP5, SAT1, and ZFP36 genes as compared to the HC group (p < 0.01 or p < 0.001).

## Discussion

TB, caused by M.tb, is an infectious disease that can affect various organs and tissues in the body. Currently, apart from HIV/AIDS, TB continues to be the leading cause of death in the world [4]. The diagnostic criterion for TB is a positive sputum smear. Unfortunately, the low rate of positive sputum smears results in a significant number of patients with underlying TB being undiagnosed, leading to delays in treatment [7, 29, 30]. The molecular mechanisms underlying the pathologies of TB are difficult to elucidate. T cell-mediated adaptive immunity is essential to combat M.tb infection in people [31]. The natural and trained innate immune system has an important role to play in the fight against Mycobacterium TB [32]. Recent studies revealed that an imbalance in ferroptotic cell death can contribute to various physiological and pathophysiological processes, which are further exacerbated by an impaired immune response [33]. The discovery of the crucial role of ferroptosis in infectious diseases has brought attention to its potential as a therapeutic target. Therefore, it is widely anticipated that it will be developed as a new therapeutic strategy for infectious diseases [34]. A recent study suggested that ferroptosis is an important necrotic mechanism in Mtb infection and a target for host-targeted treatment of TB [17]. Thus, identifying ferroptosis-related markers in TB patients is clinically important for developing new targets for diagnosis, treatment and prognosis.

High-throughput technique and microarray technology have become essential tools for studying gene expression levels and identifying the underlying molecular mechanisms associated with complex diseases [35, 36]. In the present study, a total of 22 FRGs showed differential expression between the TB and HC groups using bioinformatics techniques to analyse the GSE83456 dataset. According to the WGCNA results, three of these FRGs were identified as core diagnostic biomarkers involved in regulating the immune and inflammatory response in TB. The CHMP5, SAT1, and ZFP36 genes were all closely correlated with immune cell infiltrations. Charged multivesicular body protein 5 (CHMP5) is an anti-apoptotic protein that is thought to play a role in leukaemogenesis [37, 38]. The protein CHMP5 plays a role in regulating NF-κB signaling after RANK activation in osteoclasts [39]. CHMP5 deficiency is likely to activate programmed cell death pathways [40]. Spermidine/Spermine N1-acetyltransferase 1 (SAT1) is a crucial enzyme in the global regulation of polyamine catabolism. Its primary function is to catalyze the acetylation of spermine and spermidine, resulting in the formation of N1-acetylspermine and N1-acetylspermidine, respectively [41]. A previous study uncovered a metabolic target of p53, SAT1, which plays a role in the p53-mediated response to reactive oxygen species and ferroptosis [42]. SAT1 expression significantly correlated with infiltrating macrophages and CD8 + T cells in low-grade glioma [43]. The knockout of the zinc finger protein 36 (ZFP36) gene demonstrated the essential function of tristetraprolin in regulating inflammation [44]. A recent research has uncovered that members of ZFP36 contribute to the inflammatory features of dermal fibroblasts [45]. Previous studies have revealed the significant role of ZFP36 RNA-binding proteins in controlling T cell expansion and effector functions. Consequently, inhibiting ZFP36 could be a promising approach to improve immune-based therapies [46]. In the present study, the results of GSEA and immune cell infiltration suggested that these genes may play a crucial role in regulating the immune response in TB.

In our study, we extracted the expression matrix of 22 FRGs and calculated ferroptosis score for TB patients using the GSVA method to identify heterogeneity among TB patients. The patients with TB were separated into two ferroptosis-related subgroups (HF and LF). Based on the GSVA results, it was observed that the HF subgroup exhibited activation of immune and inflammation-related pathways, including immune system development and TNFA signaling via NFKB. To gain a better understanding of the relationship between TB subgroups and immunity, the immune landscape between subgroups was further investigated. We observed the CD4 + memory T cells, CD4 + naive T cells, CD4 + T cells, CD8 + T cells, CD8 + Tem, macrophages, macrophages M1, mast cells, monocytes, neutrophils, NK cells, Tgd cells, and Th1 cells were significantly up-regulated in the HF subgroup compared with the LF subgroup. Macrophages are the primary host cells for Mycobacterium tuberculosis during its intracellular survival in the human body [47]. Numerous studies have demonstrated that the phenotype of macrophages involved in the early stages of TB infection and granuloma formation plays a crucial role in disease progression and infection outcome [48–50]. The activity of mast cells is involved in the maintenance of a healthy lung and helps to defend against a wide range of respiratory pathogens, including Mycobacterium tuberculosis [51]. Neutrophils have been identified as a potential early indicator of TB severity, making them a promising target for host-directed therapy in TB [52]. Th1 cells have been demonstrated to aid in the protection against TB by producing IFN-γ and stimulating the antimycobacterial response in macrophages [53]. In humans, the control of Mycobacterium TB infection relies heavily on the adaptive immune response facilitated by T cells [31]. These phenomena suggested that inflammation and immunity may be involved in contributing to ferroptosis in TB patients.Additional file: As per journal requirements, every additional file must have a corresponding caption. In this regard, please be informed that the caption of Additional file [2] was taken from the additional e-file itself. Please advise if action taken appropriate and amend if necessarycorrectly

## Conclusion

Our research emphasized the significant impact of ferroptosis in the development of TB. We have identified three FRGs that may act as potential biomarkers and treatment targets for TB patients. Additionally, two molecular subtypes of TB were identified based on FRGs. Further analysis showed that dysregulation of the immune microenvironment may induce ferroptosis, thereby accelerating the progression of TB. These findings have the potential to improve diagnosis and treatment outcomes for individuals suffering from TB.

### Supplementary Information

**Additional file 1: Figure S1.** Differential expressed and functional enrichment analyses between HC and TB groups. **A** Volcano plot exhibited the differentially expressed genes (DEGs) between HC and TB groups. **B** Functional enrichment analysis of DEGs based on Metascape Online. **Figure S2.** Scatter plot exhibiting the correlation between HC phenotype and genes in royalblue module **A** and brown module **B**.

**Additional file 2: Table S1.** Sequences of primers used quantitative real-time PCR (qRT-PCR). **Table S2.** Identification of DEGs in TB patients. **Table S3.** Ferroptosis-related genes. **Table S4.** Identification hub modules by WGCNA.

## Data Availability

All data used in the present study were available from the GEO database (https://www.ncbi.nlm.nih.gov/geo/).
