# Supplementary material for: Bioinformatics-led discovery of ferroptosis-associated diagnostic biomarkers and molecule subtypes for tuberculosis patients
Source: Eur J Med Res. 2023 Oct 19;28:445. doi: 10.1186/s40001-023-01371-5 (PMC10585777; doi:10.1186/s40001-023-01371-5)
Supplement: Supplementary file 1 — Additional file 1: Figure S1. Differential expressed and functional enrichment analyses between HC and TB groups. A Volcano plot exhibited the differentially expressed genes (DEGs) between HC and TB groups. B Functional enrichment analysis of DEGs based on Metascape Online. Figure S2. Scatter plot exhibiting the correlation between HC phenotype and genes in royalblue module A and brown module B. [file 40001_2023_1371_MOESM1_ESM.docx]

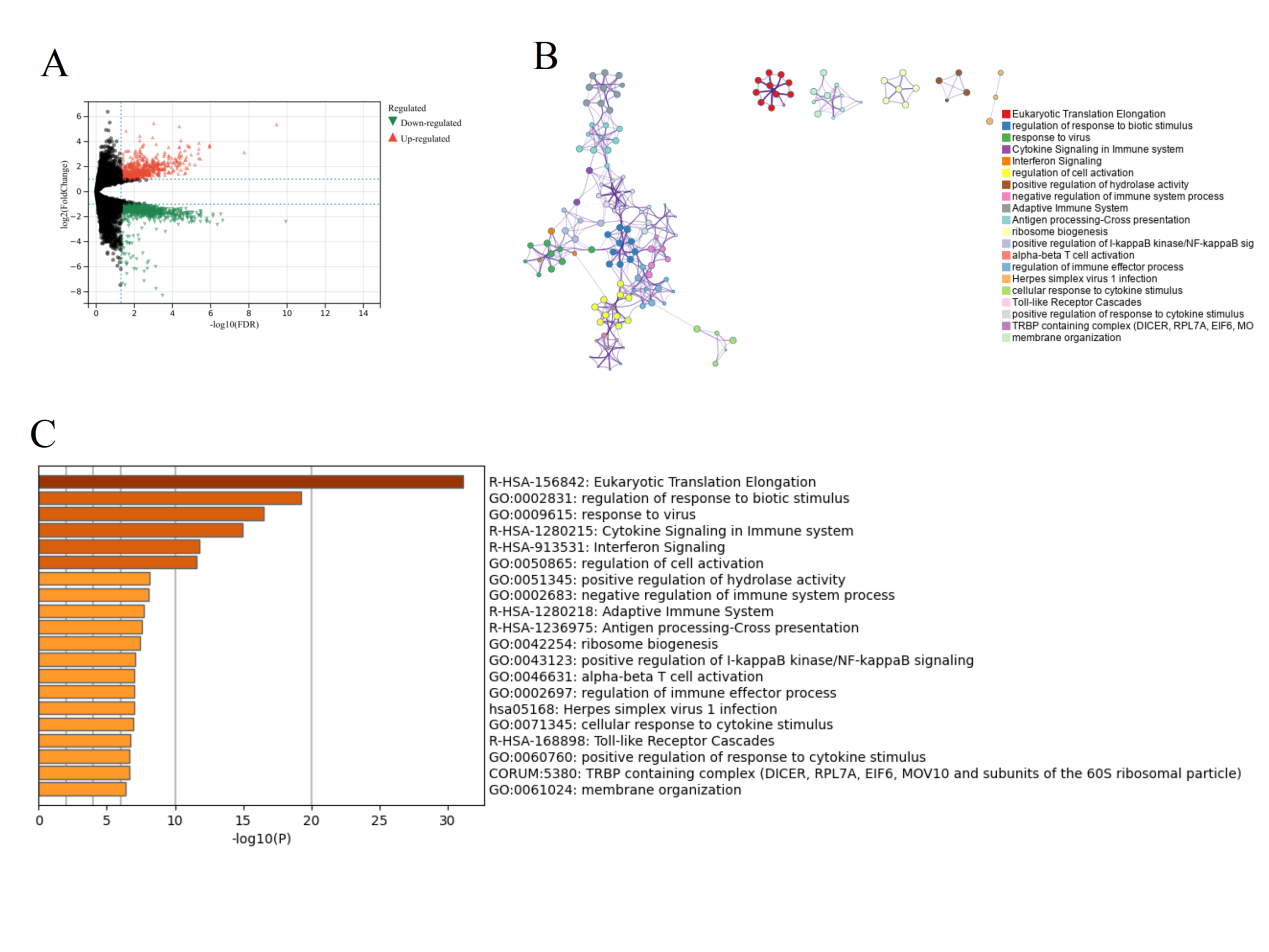


Figure S1 Differential expressed and functional enrichment analyses between HC and TB groups. (A) Volcano plot exhibited the differentially expressed genes (DEGs) between HC and TB groups. (B) Functional enrichment analysis of DEGs based on Metascape Online.


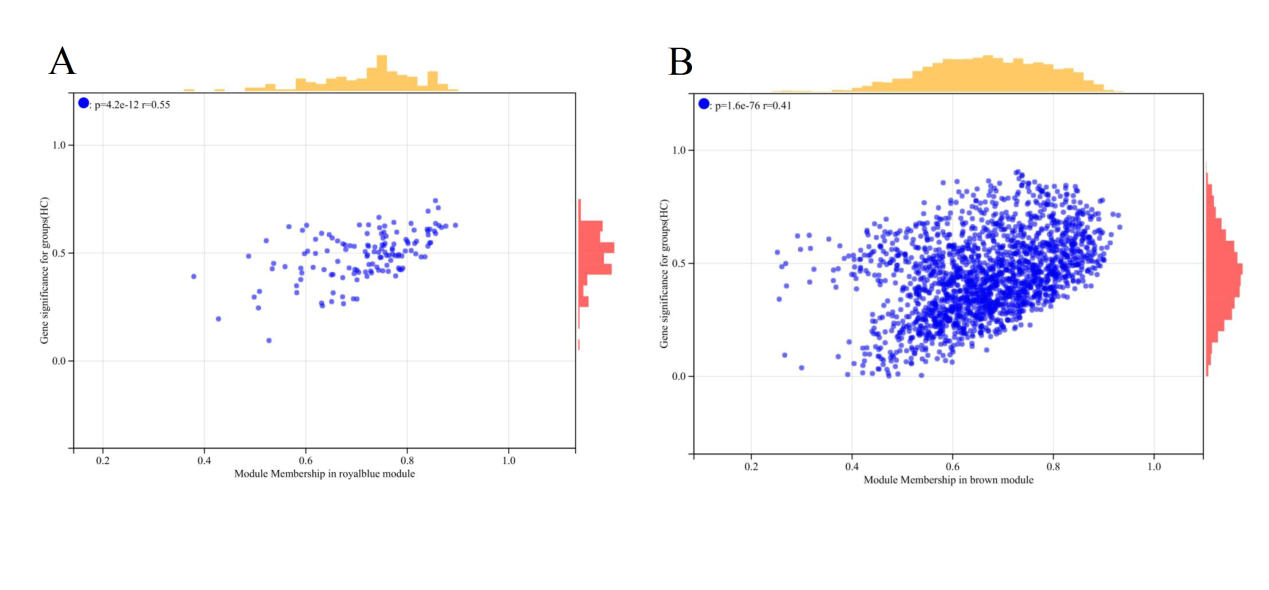


Figure S2 Scatter plot exhibiting the correlation between HC phenotype and genes in royalblue module (A) and brown module (B).
